# Supplementary material for: Complete Chloroplast Genomes of Three Medicinal Alpinia Species: Genome Organization, Comparative Analyses and Phylogenetic Relationships in Family Zingiberaceae
Source: Plants (Basel). 2020 Feb 24;9(2):286. doi: 10.3390/plants9020286 (PMC7076362; doi:10.3390/plants9020286)
Supplement: Supplementary file 1 [file plants-09-00286-s001.zip › Supplementary files/Figure S1.docx]

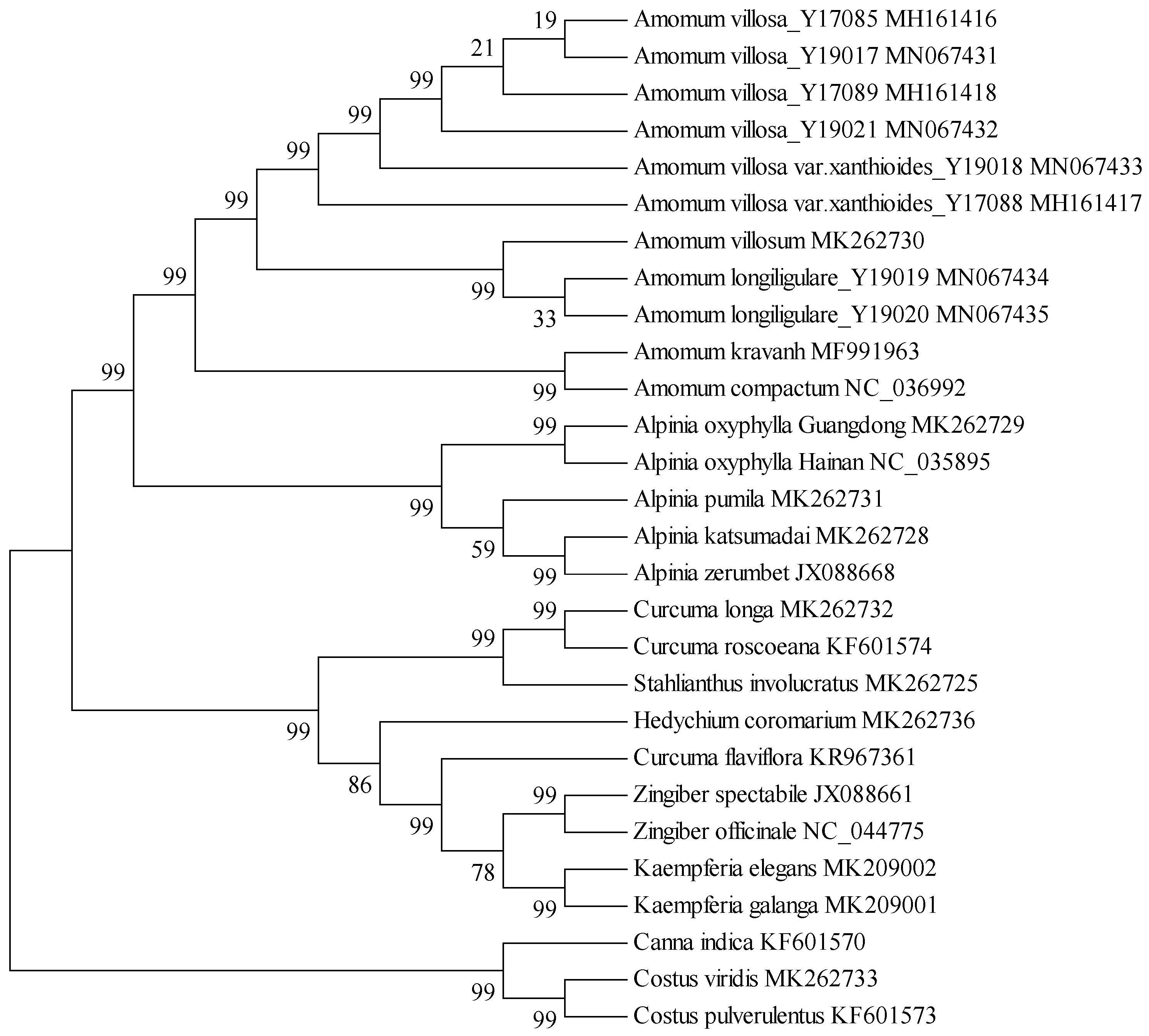


*Amomum*

Zingiberaceae

*Alpinia*

*Curcuma*Ⅱ

*Hedychium*

*Curcuma*Ⅰ

*Zingiber*

*Kaempferia*

Out group

**Figure S1**. Phylogenetic tree constructed with SNPs from 28 chloroplast genomes using maximum parsimony (MP) method. The bootstrap values were based on 1,000 replicates and were indicated next to the branches.
