# Supplementary material for: Complete Chloroplast Genomes of Three Medicinal Alpinia Species: Genome Organization, Comparative Analyses and Phylogenetic Relationships in Family Zingiberaceae
Source: Plants (Basel). 2020 Feb 24;9(2):286. doi: 10.3390/plants9020286 (PMC7076362; doi:10.3390/plants9020286)
Supplement: Supplementary file 1 [file plants-09-00286-s001.zip › Supplementary files/Table S1 Features of the chloroplast genomes of three Alpinia species..docx]

**Table S1. Features of the chloroplast genomes of three *Alpinia* species.**

| **Species** | **Regions** | **Positions** | **Length (bp)** | **T/U (%)** | **C (%)** | **A (%)** | **G (%)** | **AT/U (%)** |
| --- | --- | --- | --- | --- | --- | --- | --- | --- |
| *A.katsumadai* | Genome |  | 162,387 | 32.24 | 18.37 | 31.61 | 17.78 | 63.85 |
|  | LSC |  | 87,667 | 33.81 | 17.31 | 32.36 | 16.52 | 66.18 |
|  | IRa |  | 29,707 | 28.80 | 19.89 | 29.99 | 21.32 | 58.78 |
|  | SSC |  | 15,306 | 34.32 | 15.77 | 35.86 | 14.05 | 70.18 |
|  | IRb |  | 29,707 | 28.80 | 19.89 | 29.98 | 21.32 | 58.78 |
|  | Protein-coding genes |  | 82,998 | 31.61 | 17.17 | 31.50 | 19.72 | 63.12 |
|  |  | 1st position | 27,666 | 23.95 | 18.24 | 31.47 | 26.34 | 55.42 |
|  |  | 2nd position | 27,666 | 32.46 | 20.06 | 30.11 | 17.38 | 62.57 |
|  |  | 3rd position | 27,666 | 38.43 | 13.20 | 32.94 | 15.43 | 71.36 |
|  | tRNA |  | 2,886 | 24.95 | 23.70 | 22.04 | 29.31 | 46.99 |
|  | rRNA |  | 9,046 | 18.68 | 23.60 | 26.21 | 31.51 | 44.89 |
| *A.oxyphylla* Guangdong | Genome |  | 161,410 | 32.29 | 18.38 | 31.55 | 17.78 | 63.84 |
|  | LSC |  | 87,279 | 33.84 | 17.32 | 32.34 | 16.50 | 66.18 |
|  | IRa |  | 28,964 | 28.82 | 20.03 | 29.66 | 21.49 | 58.48 |
|  | SSC |  | 16,180 | 34.83 | 15.56 | 35.55 | 14.05 | 70.38 |
|  | IRb |  | 28,987 | 28.82 | 20.02 | 29.68 | 21.48 | 58.50 |
|  | Protein-coding genes |  | 82,281 | 31.65 | 17.17 | 31.41 | 19.77 | 63.06 |
|  |  | 1st position | 27,427 | 23.94 | 18.29 | 31.35 | 26.42 | 55.30 |
|  |  | 2nd position | 27,427 | 32.52 | 20.09 | 29.98 | 17.41 | 62.50 |
|  |  | 3rd position | 27,427 | 38.47 | 13.13 | 32.90 | 15.50 | 71.37 |
|  | tRNA |  | 2,885 | 24.96 | 23.71 | 22.05 | 29.29 | 47.00 |
|  | rRNA |  | 9,046 | 18.68 | 23.62 | 26.19 | 31.51 | 44.87 |
| *A. pumila* | Genome |  | 161,920 | 32.22 | 18.39 | 31.61 | 17.78 | 63.83 |
|  | LSC |  | 87,261 | 33.77 | 17.35 | 32.37 | 16.51 | 66.14 |
|  | IRa |  | 29,671 | 28.78 | 19.87 | 30.02 | 21.33 | 58.79 |
|  | SSC |  | 15,317 | 34.30 | 15.79 | 35.88 | 14.03 | 70.18 |
|  | IRb |  | 29,671 | 28.77 | 19.87 | 30.02 | 21.33 | 58.79 |
|  | Protein-coding genes |  | 83,007 | 31.59 | 17.13 | 31.54 | 19.74 | 63.13 |
|  |  | 1st position | 27,669 | 23.95 | 18.22 | 31.49 | 26.34 | 55.44 |
|  |  | 2nd position | 27,669 | 32.44 | 20.05 | 30.15 | 17.36 | 62.59 |
|  |  | 3rd position | 27,669 | 38.38 | 13.13 | 32.97 | 15.52 | 71.35 |
|  | tRNA |  | 2,886 | 24.95 | 23.70 | 22.04 | 29.31 | 46.99 |
|  | rRNA |  | 9,046 | 18.68 | 23.60 | 26.21 | 31.51 | 44.89 |
