# Supplementary material for: Complete Chloroplast Genomes of Three Medicinal Alpinia Species: Genome Organization, Comparative Analyses and Phylogenetic Relationships in Family Zingiberaceae
Source: Plants (Basel). 2020 Feb 24;9(2):286. doi: 10.3390/plants9020286 (PMC7076362; doi:10.3390/plants9020286)
Supplement: Supplementary file 1 [file plants-09-00286-s001.zip › Supplementary files/Table S3.Genes with introns in the chloroplast genomes of three Alpinia species as well as the exons and introns..docx]

**Table S3. Genes with introns in the chloroplast genomes of three *Alpinia* species as well as the exons and introns.**

| **Species** | **Gene** | **Location** | **Exon І (bp)** | **Intron І (bp)** | **Exon ІІ (bp)** | **Intron ІІ (bp)** | **Exon ІІІ (bp)** |
| --- | --- | --- | --- | --- | --- | --- | --- |
| *A.katsumadai* | *trnA-UGC* | IR | 35 | 801 | 38 |  |  |
|  | *trnI-GAU* | IR | 35 | 935 | 42 |  |  |
|  | *trnG-GCC* | LSC | 23 | 698 | 48 |  |  |
|  | *trnK-UUU* | LSC | 35 | 2653 | 37 |  |  |
|  | *trnL-UAA* | LSC | 35 | 537 | 50 |  |  |
|  | *trnV-UAC* | LSC | 37 | 602 | 38 |  |  |
|  | *rps12** | LSC/IR | 114 | - | 26 | 540 | 229 |
|  | *rps16* | LSC | 218 | 729 | 40 |  |  |
|  | *rpl2* | IR | 443 | 650 | 391 |  |  |
|  | *rpl16* | LSC | 402 | 1056 | 9 |  |  |
|  | *petB* | LSC | 6 | 850 | 648 |  |  |
|  | *petD* | LSC | 8 | 749 | 475 |  |  |
|  | *atpF* | LSC | 425 | 788 | 145 |  |  |
|  | *ndhA* | SSC | 518 | 1065 | 562 |  |  |
|  | *ndhB* | IR | 778 | 673 | 782 |  |  |
|  | *rpoC1* | LSC | 1626 | 742 | 429 |  |  |
|  | *clpP* | LSC | 252 | 625 | 306 | 843 | 60 |
|  | *ycf3* | LSC | 167 | 779 | 201 | 736 | 124 |
| *A.oxyphylla*  Guangdong | *trnA-UGC* | IR | 35 | 801 | 38 |  |  |
|  | *trnI-GAU* | IR | 35 | 935 | 42 |  |  |
|  | *trnG-GCC* | LSC | 23 | 695 | 48 |  |  |
|  | *trnK-UUU* | LSC | 35 | 2654 | 37 |  |  |
|  | *trnL-UAA* | LSC | 35 | 533 | 49 |  |  |
|  | *trnV-UAC* | LSC | 37 | 602 | 38 |  |  |
|  | *rps12 ** | LSC/IR | 114 | - | 26 | 540 | 229 |
|  | *rps16* | LSC | 218 | 731 | 40 |  |  |
|  | *rpl2* | IR | 391 | 650 | 443 |  |  |
|  | *rpl16* | LSC | 402 | 1048 | 9 |  |  |
|  | *petB* | LSC | 6 | 805 | 648 |  |  |
|  | *petD* | LSC | 8 | 749 | 475 |  |  |
|  | *atpF* | LSC | 425 | 785 | 145 |  |  |
|  | *ndhA* | SSC | 518 | 1058 | 562 |  |  |
|  | *ndhB* | IR | 778 | 673 | 782 |  |  |
|  | *rpoC1* | LSC | 1626 | 744 | 429 |  |  |
|  | *clpP* | LSC | 252 | 627 | 306 | 841 | 60 |
|  | *ycf3* | LSC | 167 | 784 | 201 | 737 | 124 |
| *A.pumila* | *trnA-UGC* | IR | 35 | 801 | 38 |  |  |
|  | *trnI-GAU* | IR | 35 | 935 | 42 |  |  |
|  | *trnG-GCC* | LSC | 23 | 701 | 48 |  |  |
|  | *trnK-UUU* | LSC | 35 | 2626 | 37 |  |  |
|  | *trnL-UAA* | LSC | 35 | 532 | 50 |  |  |
|  | *trnV-UAC* | LSC | 37 | 602 | 38 |  |  |
|  | *rps12 ** | LSC/IR | 114 | - | 26 | 540 | 229 |
|  | *rps16* | LSC | 218 | 730 | 40 |  |  |
|  | *rpl2* | IR | 391 | 650 | 443 |  |  |
|  | *rpl16* | LSC | 402 | 1046 | 9 |  |  |
|  | *petB* | LSC | 6 | 800 | 648 |  |  |
|  | *petD* | LSC | 8 | 750 | 475 |  |  |
|  | *atpF* | LSC | 425 | 787 | 145 |  |  |
|  | *ndhA* | SSC | 518 | 1058 | 562 |  |  |
|  | *ndhB* | IR | 778 | 673 | 782 |  |  |
|  | *rpoC1* | LSC | 1626 | 746 | 429 |  |  |
|  | *clpP* | LSC | 252 | 628 | 306 | 844 | 60 |
|  | *ycf3* | LSC | 153 | 808 | 228 | 710 | 132 |

* The *rps12* gene is divided into 5′-*rps12* in the LSC region and 3′-*rps12* in the IR region.
